# Impact of celiac disease in Behçet’s syndrome patients: a study based on the database of Türkiye

**DOI:** 10.55730/1300-0144.5815

**Published:** 2024-05-07

**Authors:** Nuray YILMAZ ÇAKMAK, Naim ATA, Serdar Can GÜVEN, Emin GEMCİOĞLU, Mustafa Mahir ÜLGÜ, Şuayip BİRİNCİ

**Affiliations:** 1 Department of Internal Medicine Sciences, Faculty of Medicine, Yıldırım Beyazıt University, Ankara, Turkiye; 2 Department of Strategy Development, Republic of Türkiye Ministry of Health, Ankara, Turkiye; 3 Division of Rheumatology, Department of Internal Medicine Sciences, Ankara Bilkent City Hospital, Ankara, Turkiye; 4 Department of Internal Medicine Sciences, Faculty of Medicine, Health Sciences University, Ankara, Turkiye; 5 General Directorate of Health Information System, Republic of Türkiye Ministry of Health, Ankara, Turkiye; 6 Deputy Minister, Republic of Türkiye Ministry of Health, Ankara, Turkiye

**Keywords:** Behçet’s syndrome, celiac disease, clinical features, inflammatory bowel disease, fibromyalgia, osteoporosis, early disease onset

## Abstract

**Background/aim:**

Our primary aim was to investigate the effects of concomitant celiac disease (CD) on the clinical characteristics of Behçet’s syndrome (BS) patients.

**Materials and method:**

The study was a retrospective, nationwide, multicenter study. Turkish Ministry of Health National Electronic Database (e-Nabız) is used under Health Ministry’s supervision to extract the subject’s data.

**Statistical analysis:**

Statistical analyses were made by the Statistical Package for Social Sciences (SPSS) software version 20 (IBM Corp., Armonk, New York). Continuous variables were presented by mean ± standard derivation (SD) or median (min–max) according to normality and compared by student-t test. A binary logistic regression analysis was performed to further investigating the relation between having a concomitant CD with each BD manifestation and comorbidity, frequencies of which were detected to be significantly different in the student–test.

**Results:**

A total of 84,241 patients diagnosed with BS were analyzed, and CD was identified in 175 (0.21 %) patients. The group with CD had a mean age of 41.30 ± 13.69 which was significantly younger. the prevalence of females was significantly higher (71.4%). The mean age of first admission for BS was also significantly younger in the group with CD (36.64 ± 13.28). BS patients with CD had a significantly higher prevalence of inflammatory bowel disease (27.2% vs. 7.3%, p < 0.001). When comorbid conditions were investigated depression (35.4% vs. 23.3%, p < 0.001), migraine (7.4 % vs. 2.6%, p < 0.001), fibromyalgia (10.9% vs. 4.5%, p < 0.001) and osteoporosis (12.6% vs. 6.6%, p = 0.001) were significantly more frequent in BS patients with CD.

**Conclusion:**

Our results suggest coexistence of CD in BS patients is related to female dominance and probably to an earlier disease onset. Several CD-related comorbidities as well as inflammatory bowel disease were more frequent in the CD group which implied an increased overall disease burden.

## 1. Introduction

Behçet’s syndrome (BS) is a systemic vasculitis characterized by recurrent oral and genital ulcers accompanied by various other organ manifestations comprising eye, musculoskeletal, vascular, neurologic, gastrointestinal, and cutaneous involvements. The etiopathogenesis is multifactorial, comprising factors such as genetic and environmental, and is yet to be fully clarified. BS has different prevalence rates worldwide, with a higher prevalence observed in Eastern Asia and the Middle East. The symptoms of the disease typically begin in early adulthood and can significantly impact the quality of life of affected individuals [1].

Celiac disease (CD) is an autoimmune enteropathy characterized by infiltration of the small intestine epithelium with chronic inflammatory cells and villous atrophy, due to an aberrant immune response to gluten-containing foods. It is an immune-mediated condition associated with systemic symptoms related to malabsorption and immune activation. The exclusion of gluten from the diet leads to the resolution of symptoms and enteropathy in most patients. Increased awareness and the development of serological tests have led to an increase in the incidence of the disease and a change in the distribution of clinical features. The prevalence of CD in the general population is estimated to be between 1.4%, with an increasing incidence over the years [2,3].

Behçet’s syndrome and CD are both immune-mediated conditions with complex pathogenesis. Both diseases were mediated by major histocompatibility complex genes known as human leukocyte antigen (HLA) genes. However, in BS class I genes are dominant (HLA-B5) while in CD class II (HLA-DQ2, DQ8). Nevertheless, it is intriguing whether a relation or an intersection is present between two conditions and there is limited data regarding the potential relationship between BS and CD, particularly regarding the impact of CD among individuals with BS [4–7].

This study aims to provide knowledge to further elucidate this issue by presenting results from a nationwide, big data analysis of the CD among individuals diagnosed with BS and to investigate effects of CD on the clinical scenario.

## 2. Materials and methods

### 2.1. Study design

The study was a retrospective, observational, nationwide cohort study. Turkish Ministry of Health National Electronic Database (e-Nabız) is used under Health Ministry’s supervision to extract the subjects’ data. The e-Nabız system was established by the Health Ministry in 2015 as a national health information system, to which only authorized individuals and institutions have access, which has wide bandwidth and covers all of the country. This study was carried out following the permission of Ministry of Health issue numbered 95741342-020. E-Nabız system contains the clinical records of over eighty million people in Türkiye including demographics, ever-installed ICD codes, laboratory results, drug history, and comorbidities. Ministry of Health presents services using Big Data technology, and these systems are also integrated: E-Nabız and National Healthcare Information System (NHIS)[8]. With these integrated, systems data of the patients as of January 1, 2016, up to date can be obtained.

### 2.2 Behçet’s syndrome and celiac disease diagnosis

A nationwide cohort was formed from patients with an entered ICD-10 code for BS (M35.2) twice at least 3 months apart between January 1, 2016, to December 31, 2022, who were considered to have BS. Data regarding demographics, comorbidities, major involvements other than mucocutaneous and musculoskeletal involvements and ever-used treatment agents for BS were recorded. Among the patients of this cohort, subjects with an entered ICD-10 code for CD (K90.1) at least three times and a report in the database indicating the necessity for a gluten-free diet were defined as CD patients. In addition to the data collected in the cohort, antigliadin IgA and G, antitransglutaminase IgA and G, and antiendomysium antibody test results were recorded in the CD group. Furthermore, upper gastrointestinal system endoscopy and colonoscopy results and histopathological evaluation reports were investigated, if present. Patients with a report indicating intraepithelial leukocyte infiltration, crypt hyperplasia, or villous atrophy/hypoplasia in the small intestine were considered to have a positive biopsy result for CD. Demographics, clinical characteristics, and treatment history were compared between BS patients with and without CD.

### 2.3 Comorbidities

Likewise, comorbid diseases were screened via ICD codes; for example hypertension (I10–I15), diabetes mellitus (E10–14), autoimmune hepatitis (K74,4), primary biliary cirrhosis + primary Sclerosing Cholangitis (K74,3), inflammatory bowel disease (K50, K51), fibromyalgia (M79), osteoporosis (M80), autoimmune thyroid disease(E06), liver disease (K72, K74,) etc.

## 3. Materials and methods

### 3.1. Study design

The study was a retrospective, observational, nationwide, multicenter study. Turkish Ministry of Health National Electronic Database (e-Nabız) is used under the Health Ministry’s supervision to extract the subjects’ data. The e-Nabız system was established by the Health Ministry in 2015 as a national health information system, to which only authorized individuals and institutions have access, which has wide bandwidth and covers all of the country. E-Nabız system contains the clinical records of over eighty million people in Türkiye including demographics, ever-installed ICD codes, laboratory results, drug history and comorbidities. Ministry of Health presents services using Big Data technology, and these systems are also integrated: E-Nabız and National Healthcare Information System (NHIS)[8]. With these integrated, systems data of the patients as of January 1, 2016, up to date can be obtained.

### 3.2 Behçet’s syndrome and celiac disease diagnosis

A nationwide cohort was formed from patients with an entered ICD-10 code for BS (M35.2) twice at least 3 months apart between January 1, 2016, to December 31, 2022, who were considered to have BS. Data regarding demographics, comorbidities, major involvements other than mucocutaneous and musculoskeletal involvements and ever-used treatment agents for BS were recorded. Among the patients of this cohort, subjects with an entered ICD-10 code for CD (K90.1) at least three times and a report in the database indicating the necessity for a gluten-free diet were defined as CD patients. In addition to the data collected in the cohort, antigliadin IgA and G, antitransglutaminase IgA and G, and antiendomysium antibody test results were recorded in the CD group. Furthermore, upper gastrointestinal system endoscopy and colonoscopy results and histopathological evaluation reports were investigated, if present. Patients with a report indicating intraepithelial leukocyte infiltration, crypt hyperplasia, or villous atrophy/hypoplasia in the small intestine were considered to have a positive biopsy result for CD. Demographics, clinical characteristics, and treatment history were compared between BS patients with and without CD.

### 3.3. Statistical analysis

Statistical analyses were made by the Statistical Package for Social Sciences (SPSS) software version 20 (IBM Corp., Armonk, New York). Continuous variables were presented by mean ± standard derivation (SD) or median (min–max) according to normality and compared by student-t test. Categorical variables were presented in numbers and percentages and compared by *x^2^* test. P-values <0.05 were considered significant statistically. A binary logistic regression analysis was performed further investigating the relation between having a concomitant CD with each BD manifestation and comorbidity, frequencies of which were detected to be significantly different in the student–test, presented with odd ratios (OR) with a confidence interval (CI) of 95%.

This study was conducted in accordance with the Declaration of Helsinki and approved by the Turkish Ministry of Health with a waiver of informed consent for retrospective data analysis.

Ethical considerations: This study was carried out following the permission of Ministry of Health issue numbered 95741342-020.

## 4. Results

A total of 84,241 patients diagnosed with BS were analyzed, and CD was identified in 175 patients, indicating a frequency of 0.2 %. The group with CD had a mean age of 41.30 ± 13.69, which was significantly younger. Additionally, the prevalence of females was significantly higher (71.4%). The mean age of first admission for BS was also significantly younger in the group with CD (36.64 ± 13.28) ( 1).

Common systemic manifestations other than mucocutaneous and musculoskeletal were investigated between groups. BS patients with CD had a significantly higher prevalence of inflammatory bowel disease (27.2% vs. 7.3%, p < 0.001), and a lower prevalence of uveitis (7.4% vs. 12.5%, p = 0.046). No difference was observed between the frequency of major vascular events like arterial aneurysms, pulmonary thromboembolism (PTE), and thrombosis other than PTE (Table 1). When comorbid conditions were investigated depression (35.4% vs. 23.3%, p < 0.001), migraine (7.4% vs. 2.6%, p < 0.001), fibromyalgia (10.9% vs. 4.5%, p < 0.001) and osteoporosis (12.6% vs. 6.6%, p = 0.001) were significantly more frequent in BS patients with CD. Furthermore, frequency of peripheral neuropathy, primary biliary cirrhosis and autoimmune hepatitis were also increased significantly, and the frequency of autoimmune thyroid disease increased close to significance. No significant differences were observed among common comorbidities such as hypertension, diabetes mellitus, hyperlipidemia, coronary artery disease and occurrence of malignancies (Table 1).

In terms of treatment, a gluten-free diet was implemented in all patients in the CD group. Evaluating medication treatments, both groups had similar usage of colchicine (p: 0.587). When the use of conventional immunosuppressants was considered, no significant difference was observed between groups, except for cyclosporine which was more frequent in the CD group (2.9% vs. 0.6%, p < 0.001). When biologic agents were examined, tumor necrosis factor α (TNFα) agents were more frequently administered in the CD group (Table 1).

The presence of CD was tested against other parameters to better demonstrate the relation between concomitant CD and BS manifestations and comorbidities by binary logistic regression for each parameter which was significantly different among BS patients with and without CD (Table 2). A cut-off value of 40 years of age was determined for age, age at first admission, and age at last admission for inclusion of these parameters in the logistic regression model. Our results demonstrated concomitant CD was significantly related to inflammatory bowel disease (OR: 3.347, 95% CI [2.362–4.741], p < 0.001), autoimmune hepatitis (OR: 9.512, 95% CI [1.773–51.059], p = 0.009), fibromyalgia (OR: 1.747, 95% CI [1.054–2.895], p = 0.031) and osteoporosis (OR: 1.821, 95% CI [1.120–2.960], p = 0.016) in BS patients.

All patients in the CD group underwent gastroscopy, and biopsy was performed in 134 patients out of which reports of 115 patients were obtainable and investigated. Biopsy sites were determined as 22.2% duodenum, 31.1% stomach, 46.7% stomach, and duodenum. Out of the 104 biopsy results available, CD was diagnosed positively in 74 cases. Among the patients with CD involvement according to biopsy results, patients 17 (22.9%) had concomitant inflammatory bowel disease. In the biopsy-negative group, 25 (33.7%) patients had concomitant inflammatory bowel disease (p = 0.282). Additionally, there were a total of 10 patients who had an additional colon/ileum histopathology report to stomach and/or duodenum: 4 of them had only inflammatory bowel disease findings, 1 had only CD findings, 1 had no conclusive result, and 2 had nonspecific findings that did not suggest either inflammatory bowel disease or CD. No evidence of BD intestinal findings was found in the biopsies scanned. CD-related serologic tests could be obtained in some patients and results were as follows: antigliadin IgG positivity, 26/75 (34.7%); antigliadin IgA positivity, 24/88 (27.3%); antitransglutaminase IgG positivity, 20/91 (22.0%); antitransglutaminase IgA positivity, 31/118 (26.3%); antiendomysium positivity, 25/97 (25.8%).

## 5. Discussion

In this study, CD could be identified in 175 out of 84,241 patients diagnosed and followed for BS. BS patients with CD were younger, had an earlier date for first admission for BS, and female sex was more frequent. Depression, fibromyalgia, migraine, and osteoporosis are the comorbidities increased in the CD group as well as primary biliary cirrhosis, autoimmune hepatitis, and autoimmune thyroid disease. As for systemic involvements of BS, the rate of inflammatory bowel disease was increased, and uveitis was decreased in CD patients. Logistic regression revealed CD as a risk factor for inflammatory bowel disease, autoimmune hepatitis, fibromyalgia, and osteoporosis. Ever-used treatment agents were similar except for TNFα inhibitors and cyclosporine, which were more frequently used in the CD group.

Behçet’s syndrome and CD are both immune-mediated conditions. BS is a variable vessel vasculitis, predominantly affecting young adults, in which venous and arterial vasculature in all sizes can be affected [1]. In addition to characteristic vascular manifestations such as aneurysms and thrombosis, due to the involvement of small veins, major debilitating complications such as neurologic, gastrointestinal, and eye involvement may occur. CD mainly affects small intestine, characterized by autoimmune destruction of epithelium leading to symptoms like diarrhea, abdominal pain, and bloating in addition to malabsorption [2]. Furthermore, several extraintestinal manifestations and complications have been reported such as osteoporosis, anemia, fatigue, depression, migraine, abnormal liver enzymes, and infertility, presumably due to malabsorption and impaired quality of life. The concomitance of both conditions is a phenomenon, that has been investigated before, yet data regarding this issue needs to be expanded.

The estimated prevalence of CD in the general population is reported to be approximately 1.4% based on serologic positivity and 0.7% based on histopathologic confirmation [3]. In other large studies, among healthy school children in our country, the prevalence of CD was estimated to be at least 0.47% by Dalgıç et al. [9]. The biopsy-proven disease was reported to be prevalent as 1:158 (0.62%) by Ertekin et al. [10]. The estimated BS prevalence in Türkiye had been reported to be 20–421 per 100,000 [11]. BS frequency in our study is compatible with this estimation as it is approximately 100 per 100,000, as the population of Türkiye is currently near 85 million. In a study involving BS patients from our country, biopsy-proven CD was reported to be as frequent as 1.02% [4]. In our cohort, we observed a CD frequency of 0.2% among patients accepted to have BS. However, our results probably did not reflect the exact prevalence as there are several setbacks to determining a true frequency. Our primary aim was to investigate the effects of concomitant CD on clinical characteristics of BS patients. Therefore, to be able to detect CD patients efficiently, we only defined CD in coexistence of a recurrent ICD code and a medical report indicating a necessity for a gluten-free diet, which may probably lead to underdiagnosis of CD, particularly for “silent” cases. Furthermore, we were able to extract data only from 2016. As a result, the prevalence in our cohort should be interpreted with caution.

Sex predominance may vary according to geographic region and is assumed to affect male and female sexes equally with a possible slight male predominance. However, it has been clarified that male sexes associated with a severe disease course [12]. Our results demonstrated a significant female dominance in BS patients with CD, this was probably due to the fact that CD is more frequently diagnosed in women [13]. BS generally affects young adults in 3^rd^ and 4^th^ decades. In our cohort, age at the time of analysis and age at the time of first administration with an ICD code for BS were significantly lower in BS patients with CD. Although the age at the time of first administration with an ICD code for BS did not represent, the age of BS diagnosis as we could only reach data as of 2016, we believe both results imply a younger onset for BS in the presence of CD.

The CD has been related to several extraintestinal manifestations. Neuropsychiatric complications, migraine, peripheral neuropathy, epilepsy, depression, and anxiety are some manifestations to be increased in CD patients although the true pathogenic mechanism is yet to be clarified whether it is malabsorption or else [14–17]. Accordingly, both migraine and depression rates were increased in our cohort of patients with coexisting CD. Osteoporosis is another well-known complication of CD which again significantly increased in CD patients in our study [18–20]. As a part of the “autoimmune phenomenon”, several autoimmune diseases were reported to be more frequent in CD patients such as primary biliary cirrhosis, autoimmune hepatitis, autoimmune thyroid disease, atopic dermatitis and type 1 diabetes [21–27]. Likewise, frequency of autoimmune hepatitis, primary biliary cirrhosis, and autoimmune thyroid disease seemed to increase in our BS patients with CD. Last but not least, fibromyalgia was also more frequent in CD patients in our cohort. Logistic regression analysis revealed a direct relation between autoimmune hepatitis, fibromyalgia, osteoporosis, and CD. Uveitis is a well-known complication of BS. Furthermore, it has been reported that CD is also a risk factor for noninfectious uveitis [28–30]. On the contrary, we observed a lower incidence of uveitis in the CD group, yet, regression analyses revealed no significant relation. The results of our study were consistent with the literature as CD-related complications increased in BS patients with CD, potentially increasing the overall disease burden and further deteriorating quality of life.

Gastrointestinal involvement in BS resembles inflammatory bowel disease and is indistinguishable in some cases as both conditions share many clinical characteristics [1]. GI involvement was reported to be more frequent in patients of Far East origin and far less in our country [26]. In our study, we determined the presence of GI involvement or concomitant IBD in case ICD codes for IBD were entered into the system for the patient. Our methodology could not differentiate whether it was due to BS gastrointestinal involvement or CD or patients who had isolated IBD. In our cohort, inflammatory bowel disease ICD code presence was significantly more common in patients with CD. It has been reported that CD is a predisposing condition for inflammatory bowel diseases increasing the risk up to 9-fold [31–33]. Our study revealed a significant OR of 3347 for IBD ICD code presence in BS patients with CD. The coexistence of BS and CD may have caused a “double-crush” effect in these patients causing an abundant increase in the frequency of inflammatory bowel manifestations. No significant differences were observed in our study regarding major systemic involvements such as vascular and central nervous system involvements which is by a significant increase in TNFα inhibitor use in CD group, but not in cyclophosphamide use.

Our study had several limitations. This study had a retrospective and cross-sectional design, the data was obtained through ICD codes and lacks genetic assessment, which prevented the determination of causal relationships between BS and CD. A true prevalence was impossible to obtain from our study. A limitation of data integration is that it can be challenging to combine data from multiple sources due to the need for a standardized format and structure, which may not always be possible or feasible to achieve. Yet, this setback comes along with all big data studies conducted via national healthcare databases and is admissible since the magnitude of the data compensates for potential stochastic errors in data integration. Another limitation is that our results imply an increased disease burden regarding the gastrointestinal system in BS and CD concomitant patients as ICD codes for IBD were more frequent in these patients. However, our results could not differentiate whether it was due to BS gastrointestinal involvement or CD or patients who had isolated IBD. Lastly, we further emphasize that the study was not designed to document an epidemiologic analysis of BS in our country but only to evaluate the impact of CD on clinical scenarios. The endoscopic biopsy results required for the diagnosis of celiac disease in our patients are less visible compared to the literature, but we think that this is not because their CD diagnosis was incorrect, but because we could not access the gastroscopy results performed during the unrecordable period.

All in all, our results suggest coexistence of CD in BS patients is related to female dominance and probably to an earlier disease onset. Several CD-related comorbidities were higher in the CD group which implied an increased overall disease burden. GI involvement was significantly more frequent in CD group. We believe our results may enlighten the effects of CD on BS and further, prospective studies will elucidate the issue better.

## Figures and Tables

**Table 1:** Characteristics of Behçet’s syndrome patients with and without celiac disease.

	BS with CD (N = 175)	BS without CD (N = 84066)	p
Age, years, ± SD	41.30 ± 13.69	45.93 ± 15.13	<0.001
Age at initial admission, years, ± SD	36.64 ± 13.28	41.18 ± 14.87	<0.001
Age at final admission, years, ± SD	39.76 ± 13.52	44.39 ± 15.12	<0.001
Follow-up duration, days ± SD	1154.54 ± 809.70	1194.58 ± 767.12	0.492
Sex, female, N (%)	125 (71.4)	45627 (54.3)	<0.001
Mortality, nN(%)	2 (1.1)	2671 (3.2)	0.127
BS manifestations other than musculoskeletal and mucocutaneous, N (%)			
Uveitis	13 (7.4)	10478 (12.5)	0.046
Arterial aneurysm	1 (0.6)	401 (0.5)	0.852
Pulmonary thromboembolism	1 (0.6)	559 (0.7)	0.884
Thrombosis other than PTE	4 (2.3)	3630 (4.3)	0.190
Inflammatory bowel disease	45 (27.2)	6096 (7.3)	<0.001
Demyelinating CNS disease	1 (0.6)	802 (1)	0.195
Hearing loss	1 (0.6)	976 (1.2)	0.153
Comorbidities, N (%)			
Hypertension	46 (26.3)	25352 (30.2)	0.285
DM	32 (18.3)	12111 (14.4)	0.135
Hyperlipidemia	12 (6.9)	7713 (9.2)	0.298
CAD	16 (9.1)	9610 (11.4)	0.354
Coronary disease other than CAD	10 (5.7)	3488 (4.1)	0.291
CVE	10 (5.7)	4421 (5.3)	0.778
PHT	1 (0.6)	92 (0.1)	0.065
Malignancy	9 (5.1)	2976 (3.5)	0.245
Tuberculosis	1 (0.6)	133 (0.2)	0.168
Hepatitis B & C	4 (2.3)	1205 (1.4)	0.338
PBC + PSC	2 (1.1)	84 (0.1)	<0.001
Chronic liver disease	1 (0.6)	163 (0.2)	0.255
Autoimmune hepatitis	2 (1.1)	60 (0.1)	<0.001
CKD	3 (1.7)	1559 (1.9)	0.899
Urolithiasis	3 (1.7)	1866 (2.2)	0.658
Pernicious anemia	0	83 (0.1)	0.678
Autoimmune thyroid disease	3 (1.7)	578 (0.7)	0.099
ITP	0	47 (0.1)	0.775
Depression	62 (35.4)	19546 (23.3)	<0.001
Fibromyalgia	19 (10.9)	3811 (4.5)	<0.001
Migraine	13 (7.4)	2225 (2.6)	<0.001
Asthma	18 (10.3)	6992 (8.3)	0.333
COPD	3 (1.7)	2464 (2.9)	0.346
Peripheral neuropathy	11 (6.3)	2931 (3.5)	0.042
Dementia	1 (0.6)	456 (0.5)	0.954
Glomerulonephritis	0	20 (0.0)	0.839
Osteoporosis	22 (12.6)	5561 (6.6)	0.001
Alopecia areata	0	128 (0.2)	0.606
Abortus	0	102 (0.1)	0.646
Treatment agents ever used for BS, n (%)			
Cyclophosphamide	1 (0.6)	238 (0.3)	0.470
MMF & MPA	2 (1.1)	1085 (1.3)	0.869
AZA	52 (29.7)	23893 (28.4)	0.669
CYC A	5 (2.9)	539 (0.6)	<0.001
Interferon	0	308 (0.4)	0.424
Colchicine	142 (81.1)	69903 (83.2)	0.587
IFX	13 (7.6)	2632 (3.1)	0.001
ADA	12 (6.9)	2741 (3.3)	0.007
ETN	5 (2.9)	539 (0.6)	<0.001
GOL	4 (2.3)	271 (0.3)	<0.001
CTZ	2 (1.1)	479 (0.6)	0.311
TCZ	0	217 (0.3)	0.502
Canakinumab	0	104 (0.1)	0.642

BS: Behçet’s syndrome, CD: celiac disease, PTE: pulmonary thromboembolism, CNS: central nervous system disease, DM: diabetes mellitus, CAD: coronary artery disease, CVE: cerebrovascular event, PHT: pulmonary hypertension, CKD: chronic kidney disease, ITP: immune thrombocytopenic purpura, COPD: chronic obstructive pulmonary disease, PBC + PSC Primary biliary cirrhosis+ Primary Sclerosing Cholangitis, MMF: mycophenolate mofetil, MPA: mycophenolic acid, AZA: azathioprine, CYC A: cyclosporine A, IFX: infliximab, ADA, adalimumab, ETN: etanercept, GOL: golimumab, CTZ: certolizumab, TCZ: tocilizumab

**Table 2:** Results of logistic regression analyses.

BS manifestation or comorbidity	Impact of presence of CD	
	OR [CI]	p
Uveitis	0.615 [0.347–1.091]	0.096
Inflammatory bowel disease	3.347 [2.362–4.741]	<0.001
Primary biliary cirrhosis	3.594 [0.542–23.817]	0.185
Autoimmune hepatitis	9.512 [1.773–51.059]	0.009
Autoimmune thyroid disease	1.848 [0.579–5.904]	0.300
Depression	1.290 [0.918–1.814]	0.143
Fibromyalgia	1.747 [1.054–2.895]	0.031
Migraine	1.788 [0.994–3.217]	0.052
Peripheral neuropathy	1.276 [0.643–2.535]	0.486
Osteoporosis	1.821 [1.120–2.960]	0.016

BS: Behçet’s syndrome, CD: celiac disease, OR: odds ratio
